# Potential Function-Based Molecular Dynamics Simulation of Al-Cu-Li Alloys and Comparison with Experiments

**DOI:** 10.3390/ma18112420

**Published:** 2025-05-22

**Authors:** Fei Chen, Han Wang, Yu Liu, Liangtao Qi, Quanqing Zeng

**Affiliations:** 1 School of Mechanical Engineering and Automation, College of Science and Technology, Ningbo University, Ningbo 315000, China; chenfei@nbu.edu.cn (F.C.); 2411170028@nbu.edu.cn (H.W.); 2 College of Mechanical and Electrical Engineering, Central South University, Changsha 410083, China; csuliuyu@csu.edu.cn; 3 High Performance Computing Center of Central South University, Changsha 410083, China

**Keywords:** Al-Cu-Li, molecular dynamics, multi-stage creep aging, microstructure, temperature

## Abstract

Due to their excellent specific strength and lightweight characteristics, Al-Cu-Li alloys are widely used in aerospace applications. The newly developed three-stage creep aging (CA) process ensures both the formability and high performance of the Al alloy. However, research at the atomic scale investigating the relationship between the microstructure and performance of ternary alloys under intricate heat treatment conditions remains scarce. This study investigates the microstructural evolution of Al-Cu-Li alloys during multi-stage low-high-low temperature CA experiments, combined with molecular dynamics (MD) simulations based on the neuroevolutionary machine learning potential (NEP) function. The simulation results indicate that the segregation state of lithium atoms at low temperatures is unstable and cannot persist at elevated temperatures. As the aging temperature in the second stage increases, the segregation of lithium atoms gradually diminishes. However, the low-temperature aging in the third stage facilitates continued atomic segregation, although the recovery is somewhat limited. Additionally, it was observed that high-temperature aging in the second stage reduces the material’s performance, while the low-temperature aging in the third stage contributes to the recovery of its properties. The experimental results indicate that the degree of precipitation phase enrichment decreases with the increase in temperature during the second stage but slightly increases with the low-temperature aging in the third stage. The excellent agreement between the experimental and simulation results validates the reliability of the MD simulations, providing a valuable reference for the performance enhancement and microstructural optimization of Al-Cu-Li alloys.

## 1. Introduction

With the rapid development of the aerospace industry, the demand for lightweight, high-performance alloys has been steadily increasing. As a typical heat-treatable alloy, Al-Li alloy exhibits not only low density but also high specific strength and stiffness, making it widely utilized in the aerospace field [1,2,3,4]. In particular, it plays an irreplaceable role in critical components such as aircraft structural parts, wing skins, and rocket fuel tanks. The lightweight characteristics and superior mechanical properties of Al-Li alloys significantly reduce structural weight, thereby enhancing the load efficiency and service life of aerospace vehicles. In contrast, traditional Al-Cu or Al-Zn-Mg alloys, although possessing high strength, exhibit weaker creep resistance and are prone to performance degradation at elevated temperatures. The mechanical properties of Al-Cu-Li alloys are primarily governed by their microstructural characteristics, particularly the type, morphology, distribution, and volume fraction of the precipitate phases [5]. The rich alloying elements in Al-Cu-Li alloys lead to the complexity in the types and sequence of precipitates under high-temperature conditions [6,7]. Studies have shown that among the various precipitates, the T1 phase (Al_2_CuLi) is the predominant one. Due to its high interface mismatch and unique precipitation-dislocation interaction mechanism, it contributes most significantly to the alloy’s strengthening [8,9,10]. Therefore, investigating the precipitation and dissolution of the T1 phase during the creep aging (CA) process of Al-Cu-Li alloys, as well as the underlying mechanisms, is crucial for enhancing the mechanical properties of the material while maintaining high formability.

In studies of the precipitation evolution during the CA process of Al-Li alloys, various microscopic techniques, such as transmission electron microscopy (TEM) and X-ray diffraction (XRD), are primarily employed to investigate the formation, structure, and morphological evolution of the precipitates [11,12,13]. However, experimental observation of microstructures is time-consuming and costly, leading current theoretical research to shift toward multi-scale simulation methods. Although models for atomic migration and evolution processes have been developed, existing model systems are still predominantly focused on binary alloys [14,15,16]. A commonly used simulation method is molecular dynamics (MD) simulation. MD simulations can accurately predict the dynamic processes of precipitate phases by pre-modeling, effectively overcoming the various limitations encountered in experimental studies. Numerous studies have demonstrated that MD simulations are capable of revealing the behavior of atoms within nanostructures [17]. Ouyang et al. [18] investigated the failure behavior of aluminum alloys using the MD software LAMMPS. Their simulations revealed that increasing temperature enhances atomic thermal vibrations, thereby lowering the energy barrier for dislocation motion. This facilitates dislocation multiplication and slip, increases atomic-scale disorder, and ultimately leads to a decrease in yield strength and macroscopic softening. Li et al. [19] employed MD simulations to elucidate the dynamic evolution of dislocations in Al-Zn-Mg alloys under cyclic tension, demonstrating that tuning dislocation density can markedly improve impact toughness. Tang et al. [20] utilized LAMMPS in combination with OVITO to simulate the deformation behavior of Al-Li alloys at various temperatures, finding that low-temperature pre-rolling is more conducive to the precipitation of fine T1 phases. Xiong et al. [21] adopted a Monte Carlo molecular dynamics (MCMD) approach to clarify the grain boundary segregation behavior in Al-Cu alloys. They showed that tailoring grain boundary types or introducing solute segregation, such as Al, can concurrently enhance alloy strength. Additionally, other studies have explored the nucleation mechanisms of solute clusters during the aging process of Al-Cu alloys using Monte Carlo techniques [22]. In summary, these studies have provided atomistic insights into how temperature, dislocation dynamics, and solute segregation influence microstructural evolution, laying a critical foundation for understanding the relationship between microstructure and properties during CA.

However, most existing research focuses on single-stage aging processes, whereas in practical applications, multi-stage CA with temperature modulation is emerging as a frontier in aluminum alloy heat treatment due to its capability to precisely coordinate the co-evolution of precipitate size distribution and dislocation structures. Xu et al. [23] demonstrated that introducing a low-temperature aging stage following high-temperature creep aging can effectively restore the mechanical properties of the alloy. Building upon this, Chen et al. [24] further developed a low-temperature–high-temperature–low-temperature three-stage CA process, which significantly enhances creep strain while ensuring both material strength and ductility, thereby achieving a synergistic optimization of formability and mechanical properties. However, despite the significance of Al-Cu-Li alloys in aerospace applications, there remains a notable gap in molecular-scale studies concerning their unique multi-stage creep aging behavior, particularly the precipitation kinetics of the T1 phase.

Therefore, in this study, the multi-stage creep aging process of Al-Cu-Li alloy at different aging temperatures was systematically simulated by using the MCMD simulation method, and the temperature-regulated mechanism of T1 phase precipitation kinetics was quantitatively analyzed by tracing the diffusion paths and bias behavior of Li atoms. Based on the simulation results, an optimized creep aging scheme was used for the experiments, and TEM analysis was performed on the microstructure of the tested alloys to further validate the simulation results from the experimental point of view. In addition, uniaxial tensile tests were performed on Al-Cu-Li alloys to verify the reasonableness of the mechanical properties of Al-Cu-Li determined by the neuroevolutionary machine learning potential function (NEP).

## 2. Simulation and Experimental Method

### 2.1. MD Simulation Statement

**Figure 1:**
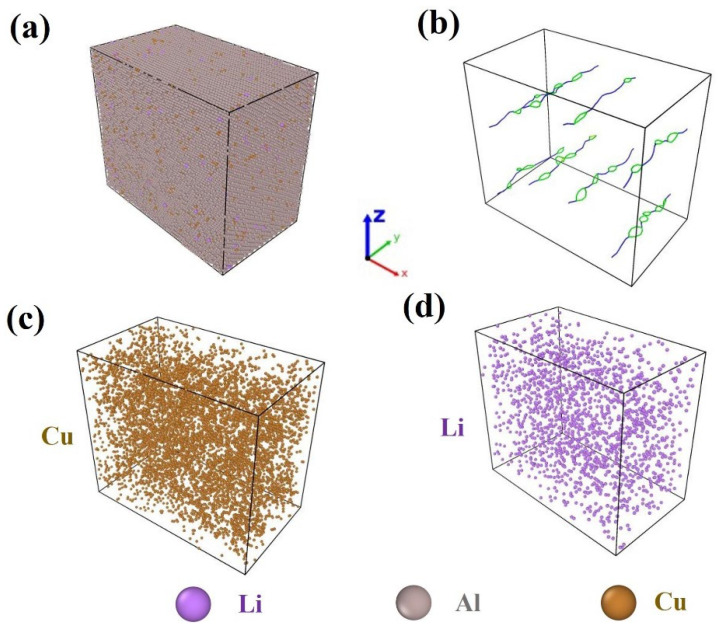
Modeling of alloy systems visualized using OVITO: (**a**) model of random solid solution and (**b**) dislocation distribution; (**c**) distribution of Cu elements in random solid solution; (**d**) distribution of Li elements in random solid solution. Adapted from Ref. [27].

In this study, MD simulations were performed using the GPUMD software (version 3.8) [25], with the dynamics visualized using OVITO (version 3.5.0) [26]. Considering the limitations of computational power, an Al-Cu-Li alloy model was constructed with dimensions of approximately 181.61 Å, 105.92 Å, and 159.78 Å in the x, y, and z directions, respectively. The model contains 181,440 atoms, with an atomic ratio of Al:Cu:Li as 95:4:1. In order to simulate the effect of dislocation defects on the precipitates, dislocations were implanted into the model, which is as shown in Figure 1. Periodic boundary conditions are used, which are based on the principle of approximating as a macroscopic system by a periodic box called a crystal cell. In this study, the neural evolutionary potential (NEP) for Al-Cu-Li alloys developed by Chen et al. [27] was employed to describe interatomic interactions. This potential has been rigorously validated, exhibiting only minor deviations from density functional theory (DFT) calculations. The root-mean-square errors for energy, atomic forces, and virial stress are 2.1 meV/atom, 47.4 meV/Å, and 14.8 meV/atom, respectively, demonstrating its excellent training accuracy. Specifically, the potential energy surface function $U_{i}$ for each atom i as realized by modeling the descriptor vector $q_{\nu }^{i}$ as follows [28]:

(1) $$U_{i}=\sum_{\mu =1}^{N_{neu}}w_{\mu }^{\left(1\right)}\tanh\left(\sum_{\nu =1}^{N_{des}}w_{\mu \nu }^{\left(0\right)}q_{\nu }^{i}-b_{\mu }^{\left(0\right)}\right)-b^{\left(1\right)}\;\;\;\;\;\;\;\;\;\;$$

where $\tanh\left(x\right)$ is the activation function of the hidden layer; $N_{des}$ is the number of components of the descriptor vector; $N_{neu}$ is the number of neurons; $w^{\left(0\right)}$ is the matrix of connection weights from the input layer to the hidden layer; $w^{\left(1\right)}$ is the vector of connection weights from the hidden layer to the output layer node $U_{i}$; $b^{\left(0\right)}$ is the bias vector of the hidden layer, and $b^{\left(1\right)}$ is the bias of the output layer node $U_{i}$.

Due to the limitation of computational resources and time cost, MD simulation can only carry out nanosecond (ns)-level time scale simulation, so it is difficult to compare with the macro-scale molding process, but it does not hinder the study of the laws of atomic motion. The MD simulation conditions and parameter settings are shown in Table 1. The simulations were performed under an NPT ensemble (constant pressure and temperature) with a time step of 1 fs. The local stress state in the central region of the specimen served as the reference for imposing loading conditions in the MD simulation. Under actual processing conditions, the specimen is subjected to uniaxial tensile stress. Due to Poisson’s effect, the internal material contracts in the directions perpendicular to the tensile axis, which induces transverse tensile stresses from the surrounding material. Consequently, the internal region experiences a triaxial tensile stress state, which was replicated in the simulation by applying tensile stresses in all three directions.

In order to simulate the effect of different stages and temperatures on the precipitation characteristics, we performed MD simulations of the solid solution with different isothermal processes individually or consecutively according to the process setup. Isothermal equilibrium process using MCMD simulation. Considering the time cost, the isothermal equilibrium below each temperature is 2 × 10^6^ steps, at which time the potential energy tends to be stabilized. Since the MD simulation cannot operate under the time scale of the real experimental level, it only relies on the MD simulation to qualitatively study the law of temperature effect precipitation to provide support for the selection of the subsequent creep process.

### 2.2. Materials

**Figure 2:**
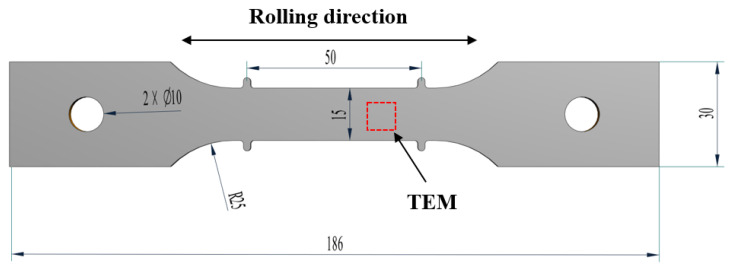
Specimen geometry and size (mm).

The raw material used in this study is a 10 mm thick Al-Cu-Li alloy plate, with its specific chemical composition presented in Table 2. This material was supplied by Southwest Aluminum (Group) Co., Ltd., in Chongqing, China. The alloy sheets were then cut along the rolling direction into standard creep samples with a thickness of 2 mm, as shown in Figure 2. Prior to the experiment, the samples underwent a pre-treatment process as follows: the samples were placed in a resistance furnace and solution-strengthened at a holding temperature of 460 °C for 40 min, with the furnace temperature variation controlled to within 3 °C. After solution treatment, the samples were rapidly quenched in cold water within 5 s, until their temperature dropped to room temperature. To prevent natural aging from affecting the material properties, the solution-treated samples were stored at −20 °C for refrigeration.

### 2.3. Creep-Aging Tests and Mechanical Properties Tests

**Figure 3:**
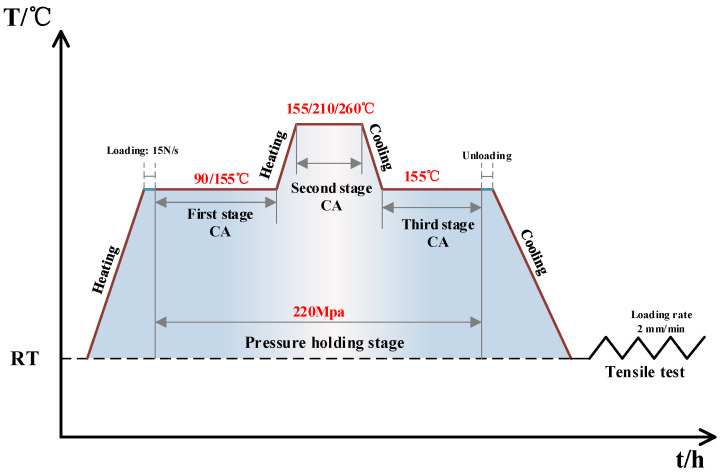
Experimental scheme of three-stage CA of 2195 aluminum-lithium alloy.

Creep tests were conducted on the solution-treated samples using the RMT-D5 electronic creep testing machine (Sansi Taijie Technology Co., Ltd., Zhuhai, China) equipped with a heating furnace. The samples were positioned at the center of the furnace’s isothermal zone, and the temperature distribution within the furnace was monitored in real time using a thermocouple system with three probes, ensuring that temperature fluctuations were maintained within ±1.5 °C. To eliminate mechanical play in the loading system, a pre-load of 200 N was applied axially before testing. During the creep test, a grating-type displacement sensor (Sansi Taijie Technology Co., Ltd., Zhuhai, China) was employed to accurately measure the creep strain of the specimen in real time. The arrangement of the three-stage CA test is shown in Figure 3: The sample was heated at a rate of 5 °C/min to the temperature set for the first stage of the three-stage CA process (90/155 °C), where it was held. Subsequently, the tensile stress on the sample was increased to 220 MPa and maintained. Once the stress-holding time reached the set duration for the first stage of the CA process, the sample was further heated to the required temperature for the second stage (155/210/260 °C) and held for the specified time. Subsequently, the temperature was decreased to reach the third stage of the CA temperature, where it was held until the completion of the three-stage CA experiment, after which the sample was removed. In addition, tensile tests were conducted at room temperature using a 100-KN CMT-5000 testing machine (Sansi Taijie Technology Co., Ltd., Zhuhai, China) (equipped with a YYU-12.5-25 electronic tensiometer (NCS Testing Technology Co., Ltd., Jinan, China)) at a tensile rate of 2 mm/min, with the direction of tensile being parallel to the direction of cold rolling, and each set of process parameters was repeated three times and averaged to ensure the reliability of the data.

### 2.4. Microstructure Characterization

After the CA tests, the microstructural characterization of the samples was performed using a Thermo-Fisher Talos F200X Scanning Transmission Electron Microscope (STEM) (Thermo Fisher Scientific Inc., Waltham, MA, USA) equipped with a High-Angle Annular Dark-Field (HAADF) detector (Thermo Fisher Scientific Inc., Waltham, MA, USA). Prior to TEM analysis, samples were extracted from the central region of the specimen, mechanically polished to a thickness of approximately 70 μm, and punched into discs with a diameter of 3 mm. Subsequently, they were electropolished at −30 °C using a TenuPol-5 twin-jet electrolytic polisher (Struers ApS, Ballerup, Denmark), with a solution of 30% nitric acid and 70% methanol to achieve electron transparency suitable for STEM observations.

## 3. Results and Discussion

### 3.1. MD Simulation Results of Dislocation–Precipitate Interaction

**Figure 4:**
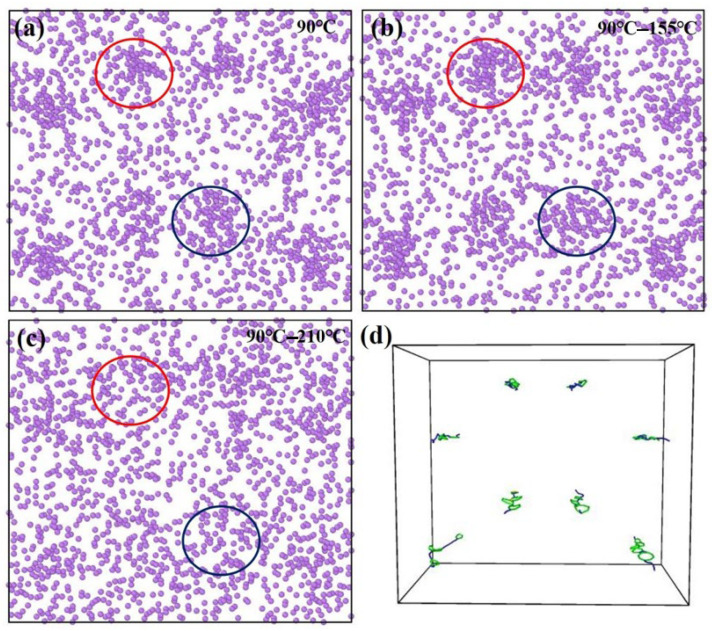
MD simulation results of Li atom aggregation structures in Al-Cu-Li alloys under different thermal processing conditions using the NEP function. (**a**) Atomic segregation observed after 90 °C; (**b**) Retained segregation after the 90–155 °C process; (**c**) Disappearance of segregation after the 90–210 °C process; (**d**) Dislocation distribution.

During the CA of Al-Cu-Li alloys, temperature increase significantly promotes dislocation slip and atomic diffusion to enhance creep strain [29]. Based on the previous study [29], this study further optimized the process parameters by increasing the second-stage aging temperature to 210 °C and constructed a 90–210 °C two-stage CA process to increase the amount of creep in the second stage. As a comparison, MD simulations were also performed for the 90 °C and 90–155 °C processes in the previous study. It has been proved that the diffusion kinetics of Li atoms is closely related to the precipitation of the T1 phase [30]; therefore, we take the change in the distribution of Li atoms to illustrate the changes and laws of the precipitation process, and the distribution of Li atoms under different processes is shown in Figure 4.

As shown in Figure 4, after isothermal equilibration at 90 °C, atomic segregation occurs at dislocation sites due to the diffusion pathways and energy provided by dislocations [31]. This observation aligns with the fact that precipitates preferentially form at dislocation sites [19,32]. Figure 4b illustrates the atomic distribution following the completion of the 90–155 °C treatment. It is evident that the segregation phenomenon persists, indicating that the temperature in the second stage (155 °C) does not disrupt the precipitate characteristics formed during the first stage (90 °C). Instead, these characteristics are preserved and continue to evolve during subsequent precipitation. Figure 4c shows the atomic distribution after increasing the temperature of the second stage from 155 °C to 210 °C, corresponding to the completion of the 90–210 °C treatment. In the circled regions, no significant segregation is observed, suggesting that the microstructure formed during the first stage of CA at 90 °C is unable to persist during the subsequent aging process at 210 °C. This microstructure is rapidly dissolved, indicating that microstructures formed at low temperatures are thermodynamically unstable and cannot persist at excessively high temperatures. Therefore, after the creep temperature of the second stage has been increased, the creep temperature of the first stage must also be increased to ensure that the microstructure at the low temperature of the first stage will be stable enough to continue to precipitate or not dissolve significantly at the subsequent high temperature of the second stage.

**Figure 5:**
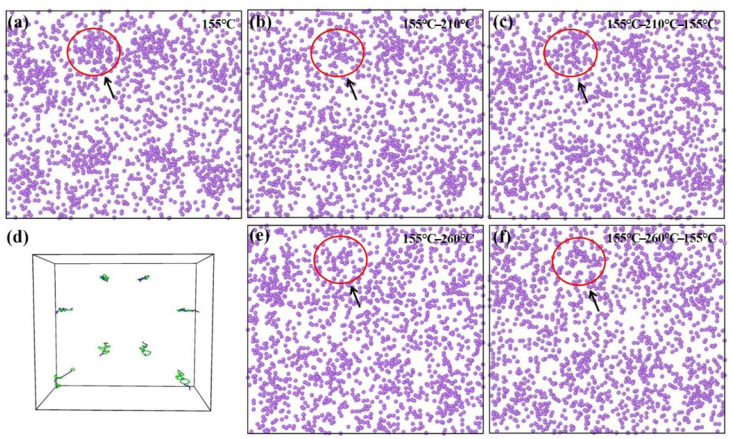
MD simulation results of Li atom segregation structures in Al-Cu-Li alloys under different thermal processing conditions using the NEP function. (**a**) Pronounced atomic segregation at 155 °C; (**b**) Slight dissolution of the segregation structure during the 155–210 °C process; (**c**) Similar to (**b**), weak atomic segregation after the 155 °C-210 °C-155 °C cycle; (**d**) Dislocation distribution; (**e**) Disappearance of atomic segregation during the 155–260 °C process; (**f**) Retained atomic dispersion after the 155 °C-260 °C-155 °C cycle.

The aging performance data provided by the Al-Li alloy raw material manufacturer indicates that the alloy exhibits superior overall mechanical properties at 155 °C, suggesting that the microstructural state of the material at this temperature is relatively optimal. Consequently, the first-stage creep temperature is set to 155 °C in order to provide an ideal microstructural condition for the subsequent creep process. Figure 5a shows the distribution of Li atoms after isothermal equilibrium at a temperature of 155 °C, and Figure 5b shows the distribution of Li atoms after the end of the process at 155–210 °C. After the comparison of the two figures, it can be found that the second stage at a temperature of 210 °C basically maintains the precipitation state formed in the first stage (155 °C), although the polarized region shows a weak dissolution trend. When the temperature of the second stage is increased to 260 °C, as shown in Figure 5e, the dissolution of the segregation regions becomes more pronounced, and a distinct difference from the precipitate state formed during the first stage (155 °C) is observed. This evidence confirms that, for the creep process at the first stage temperature of 155 °C, the second stage creep temperature should not exceed 260 °C. The increase in temperature increases the creep strain, while the disappearance of segregation indicates the re-dissolution of precipitates, which in turn signifies a weakening of precipitation strengthening. This inevitably results in a reduction in strength. To enhance strength once more, a third stage of low-temperature creep was introduced following the second stage of high-temperature creep. Similarly, the third-stage creep temperature was set to 155 °C to allow for the improvement of the microstructure once again. The atomic distribution resulting from the two sets of three-stage creep processes, 155 °C-210 °C-155 °C and 155 °C-260 °C-155 °C, is shown in Figure 5c,f. It is evident from both figures that atomic segregation has partially recovered (continuing precipitation or secondary precipitation), which contributes to the restoration and enhancement of strength. The atomic segregation in Figure 5c shows little change compared to that in Figure 5b. However, in Figure 5f, atomic segregation is not distinctly observed, indicating that the temperature of the second high-temperature stage (260 °C) suppresses atomic segregation during the subsequent third-stage low-temperature creep process. This suggests that different temperatures in the second high-temperature stage have distinct mechanisms influencing subsequent precipitation/segregation.

**Figure 6:**
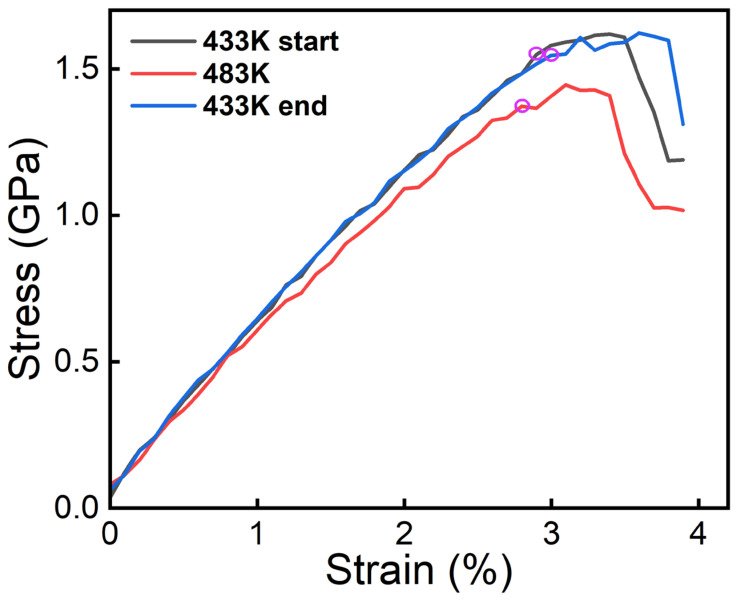
Comparison of the strength of Al-Cu-Li alloys at different stages in the multi-stage CA process.

The above analysis indicates that when the temperature of the second high-temperature creep stage is increased to 210 °C, 90 °C is no longer an optimal choice for the first-stage creep temperature. A first-stage creep temperature of 155 °C is more suitable. Additionally, the temperature of the high-temperature creep stage should not exceed 260 °C. The third-stage low-temperature creep process (155 °C) can restore the dissolution caused by the high-temperature stage. To verify whether the third-stage low-temperature aging phase leads to a recovery in strength, MD simulations were performed using the NEP function. The entire model consists of approximately 181,440 atoms, and strength simulations were conducted using the NVT ensemble with a Velocity-velocity stretching method, setting the strain rate to $10^{8}s^{-1}$. The strength simulations were carried out according to the different stages of the multi-stage CA process, with the 155 °C-210 °C-155 °C process used as an example, corresponding to 428 K-483 K-428 K. The gray curve in Figure 6 represents the stress-strain curve of the alloy during the first stage (155 °C); the red curve represents the stress-strain curve during the second stage (155–210 °C); and the blue curve represents the stress-strain curve during the third stage (155 °C-210 °C-155 °C).

The curves in the figure indicate that the alloy exhibits the highest strength during the first stage, a reduction in strength due to high temperatures in the second stage, and a subsequent increase in strength during the third stage. This behavior is associated with the dissolution and secondary precipitation of precipitates. As validated in the MD simulations in the previous section, the high temperatures in the second stage cause precipitates to dissolve, leading to a decrease in strength, while the low temperatures in the third stage facilitate secondary precipitation of precipitates, enhancing strength.

As shown in Figure 6, the tensile strength predicted by MD simulations is significantly higher than the experimentally measured strength. This discrepancy, at the mechanistic level, primarily stems from inherent limitations in the scale and idealization assumptions of MD simulations. Specifically, atomistic models typically represent defect-free single-crystal systems and neglect mesoscale microstructural features, such as grain boundaries and phase interfaces. These structures are prevalent in real polycrystalline materials and often act as stress concentrators and dislocation sources, thereby facilitating plastic deformation and markedly reducing the macroscopic mechanical strength. In addition, the simplified ternary Al–Cu–Li system used in the simulations omits trace alloying elements commonly present in commercial alloys, such as Mg, Zn, and Fe. Although these elements exist in minor quantities, they exert non-negligible effects on precipitation thermodynamics, solute drag, and vacancy migration. For example, Mg has been shown to synergistically promote the formation of the S′ phase with Cu [33], while Zn may influence the nucleation kinetics of the T1 phase [34]. Neglecting these elements may lead to an overestimation of the coherency or thermal stability of the precipitates, which in turn exaggerates the resistance to dislocation motion and results in an overprediction of strength in the simulations. Therefore, the absence of microstructural constraints and the idealized alloy composition collectively contribute to the strength disparity between MD simulations and experimental results.

In summary, the predictive capability of MD simulations lies mainly in capturing relative trends in performance evolution, while the absolute degree of strengthening and underlying mechanisms require experimental validation and analysis. The following sections present experimental evaluations of the proposed processing routes to identify the optimal treatment conditions.

### 3.2. Mechanical Properties and Microstructure After Multi-Stage CA

**Figure 7:**
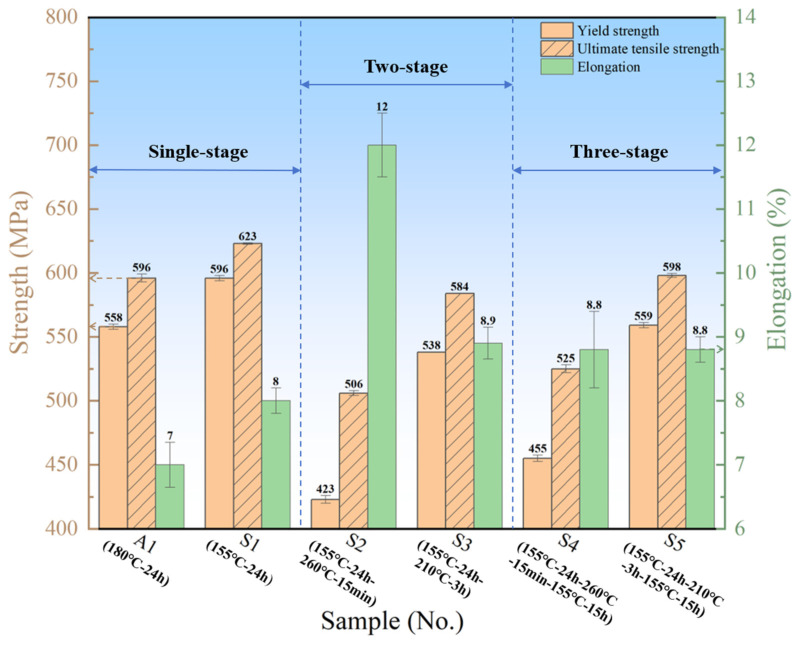
Mechanical properties of samples under different CA processes.

In order to systematically study the process characteristics of three-stage CA, a series of basic experiments were carried out, and the mechanical properties under different process conditions were compared. To facilitate the presentation of experimental results, we assigned unique identifiers to each process sample, with the specific codes and their corresponding comprehensive properties shown in Figure 7. The following analysis focuses on the mechanical properties of the specimens under different processing conditions, aiming to explore the influence of each stage on the overall mechanical properties of the specimens after the three-stage CA process. The process temperature commonly employed for the CA forming of Al-Li alloy components is 180 °C. Therefore, the mechanical strength of the current 180 °C CA process is used as the reference baseline. Process A1 (180 °C-24 h-220 MPa) represents the existing CA procedure for Al-Li alloys and serves as the benchmark for evaluating other CA processes.

Previous studies have indicated that the Al-Cu-Li alloy reaches its peak aging state after 24 h of aging at 155 °C, with no significant improvement in material properties observed upon further extension of the aging time [24]. Therefore, in this study, the aging time for the first stage is fixed at 24 h. A comparison of the performance data for specimens S1 (155 °C-24 h-220 MPa) and S2 (155 °C-24 h-260 °C-15 min-220 MPa) in Figure 7 reveals that when the aging temperature of the second stage is raised to 260 °C, a mere 15 min of aging results in a significant reduction in tensile strength, from 623 MPa to 506 MPa (an 18.8% decrease). This observation is consistent with the MD simulation results presented in Figure 5e, suggesting that the aging temperature for the second stage should not exceed 260 °C. To investigate the compensation effect of low-temperature aging, a third aging stage (155 °C-15 h) was introduced based on the S2 process, resulting in specimen S4 (155 °C-24 h-260 °C-15 min-155 °C-15 h-220 MPa). The yield strength (YS) and ultimate tensile strength (UTS) of S4 were restored to 455 MPa and 525 MPa, respectively, showing an increase of approximately 30 MPa compared to the S2 specimen. However, the elongation decreased by 3%. Further optimization of the process revealed that the S3 specimen (155 °C-24 h-210 °C-3 h-220 MPa), which was aged at 210 °C-3 h as the second stage of aging, showed a slowing down of the strength decrease (538 MPa for YS and 584 MPa for UTS) by about 60 MPa compared to the S2 specimen. Notably, the comprehensive performance of the S5 specimen (155 °C-24 h-210 °C-3 h-155 °C-15 h-220 MPa) is comparable to that of the traditional single-stage CA process A1 (180 °C-24 h-220 MPa), with YS and UTS reaching 559 MPa and 598 MPa, respectively. Additionally, the elongation increased from 7% in A1 to 8.8% in S5, indicating that the three-stage CA process not only effectively maintains the mechanical properties but also significantly enhances the plasticity of the material. Further analysis of the creep strain reveals that the creep strain of A1 is 0.149%, while that of the S5 specimen reaches 0.242%, representing a 63% increase compared to A1 [24]. This remarkable difference primarily arises from the high-temperature stage in the three-stage CA process, which accelerates atomic diffusion and dislocation motion, significantly reducing the activation energy for dislocation climb and glide. Consequently, the creep rate is substantially increased, ultimately leading to a higher accumulation of creep strain.

In summary, after systematic low-temperature single-stage, low-temperature-high-temperature two-stage, and low-temperature-high-temperature-low-temperature three-stage CA experiments, it was observed that the mechanical properties of the specimens rapidly declined after the second-stage high-temperature aging. However, the strength was restored following the third-stage low-temperature aging. This trend aligns well with the MD simulation results shown in Figure 5 and Figure 6. The observed changes are primarily attributed to the thermodynamic evolution of precipitates, with the enrichment of Li during low-temperature aging providing favorable conditions for the formation of the T1 phase [35]. The T1 phase is the primary strengthening phase in Al-Cu-Li alloys [10]. As the temperature increases, the re-dissolution of solute elements becomes more pronounced, causing the strengthening phase to either cease continuous precipitation or begin to dissolve. To validate this mechanism, TEM will be used to characterize the microstructure of the creep-aged specimens, establishing a connection between the microstructure and macroscopic properties while further confirming the MD simulation results.

**Figure 8:**
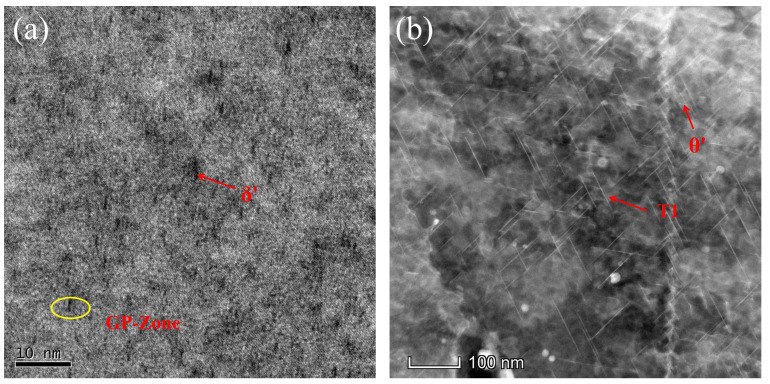
HAADF-STEM micrographs in the <110>Al zone axis of the samples. (**a**) Early-stage precipitation observed after the 90 °C-6 h-220 MPa process; (**b**) Increased precipitation density after the 90 °C-6 h-155 °C-15 h-220 MPa process.

Figure 8 illustrates the internal microstructure of the Al-Cu-Li alloy specimens before and after the second-stage high-temperature CA. A comparison between Figure 8a (90 °C-6 h-220 MPa) and Figure 8b (90 °C-6 h-155 °C-15 h-220 MPa) reveals a significant difference in precipitate density, with the latter exhibiting a more densely distributed precipitate phase. This considerable increase in the number of precipitates suggests that, despite the second stage aging temperature rising to 155 °C, the precipitate kinetics remain continuous with those of the first stage (90 °C). This observation is consistent with the simulation results shown in Figure 4b. According to Li et al., the precipitation temperature range of the T1 phase is between 120 °C and 190 °C [36]. Therefore, it can be inferred that as the second-stage aging temperature increases, the system gains a higher thermodynamic driving force, significantly accelerating the diffusion rate of solute atoms [37]. This change in kinetic conditions promotes a characteristic transformation in the microstructure, from clusters of enriched Cu and Li atoms along with the δ’ (Al_3_Li) phase to an emerging precipitate structure dominated by a small amount of θ’ (Al_2_Cu) transition phase and a substantial presence of the T1 strengthening phase. It is noteworthy that, according to previous studies [38,39], the clusters and other precipitates formed during the low-temperature stage rapidly dissolve during the high-temperature stage. This is consistent with the MD simulation results of the two-stage CA process between 90 °C and 210 °C, as shown in Figure 4c.

**Figure 9:**
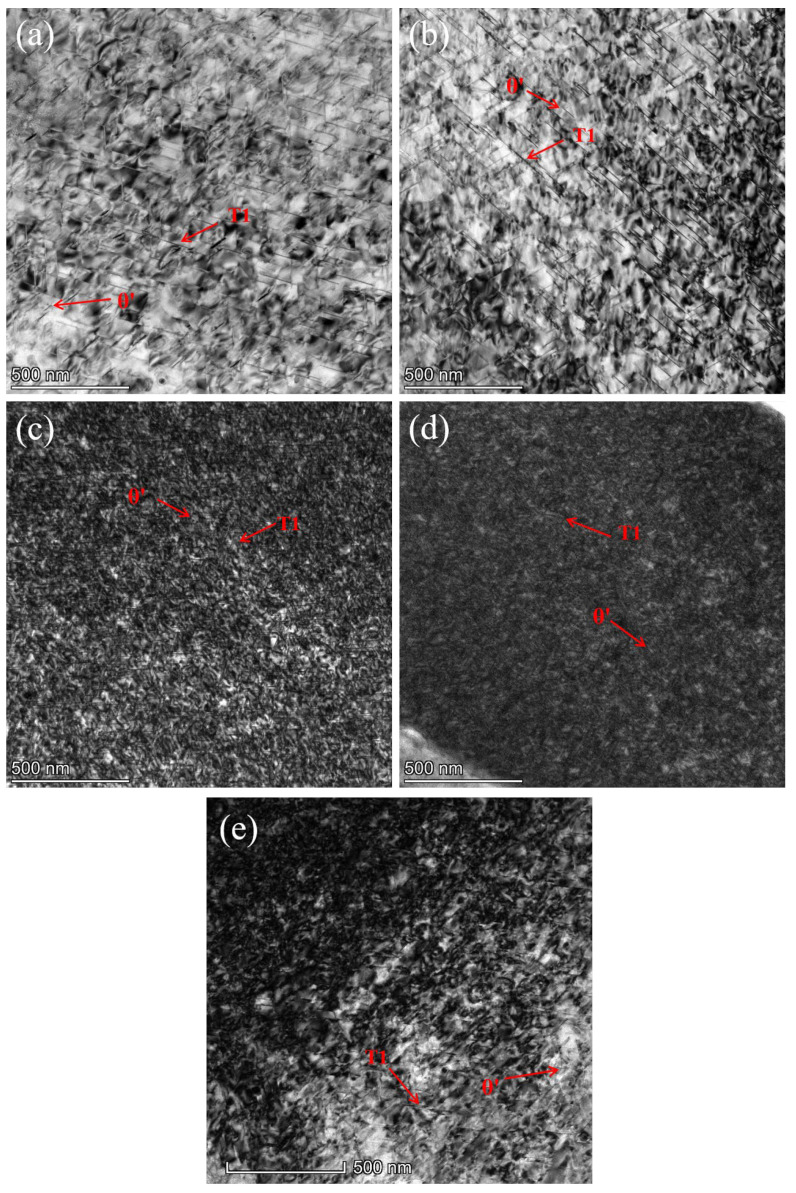
HAADF-STEM micrographs in the <110>Al zone axis of the samples. (**a**) Sparse and coarsened precipitates after the 155 °C-24 h-260 °C-15 min-220 MPa process; (**b**) Dense precipitates with secondary precipitation after the 155 °C-24 h-260 °C-15 min-155 °C-15 h-220 MPa process; (**c**) Similar precipitation morphology to (**e**), indicating stability after the 155 °C-24 h-210 °C-3 h-220 MPa process; (**d**) Dense precipitates with increased quantity after the 155 °C-24 h-210 °C-3 h-155 °C-15 h-220 MPa process; (**e**) Stable and fine precipitates after the 155 °C-24 h-220 MPa process.

By comparing the precipitate morphologies in Figure 9e (155 °C-24 h-220 MPa) and Figure 9c (155 °C-24 h-210 °C-3 h-220 MPa), no significant differences are observed, indicating that increasing the aging temperature in the first stage of CA helps maintain the thermal stability of precipitates during the high-temperature stage (210 °C), thereby suppressing the re-dissolution of precipitates. This observation is consistent with the simulation results presented in Figure 5b. By measuring the precipitate size and its distribution, it can be observed that the precipitate lengths in both Figure 9c,e are primarily concentrated within the range of 60~80 nm. Notably, Figure 9e exhibits a slightly higher number of coarse precipitates exceeding 100 nm compared to Figure 9c. This indicates that during the second-stage high-temperature aging at 210 °C, although the precipitate size shows a slight increase, the overall distribution characteristics remain relatively similar. However, when the second stage CA temperature is raised to 260 °C, as seen in Figure 9a, the precipitates become sparser but coarser compared to those in Figure 9e, with the average length reaching 125 nm, which is significantly higher than that in Figure 9e. This indicates that, although the high-temperature stage lasted only 15 min, it still caused the re-dissolution of the fine precipitates formed during the first stage, leading to an increase in the solute atom concentration within the grains, thereby providing the driving force for Ostwald ripening [40]. In summary, the second-stage creep temperature should not exceed 260 °C to avoid excessive coarsening of the T1 phase, which would otherwise degrade the material’s performance. This observation aligns with the simulation results shown in Figure 5e. To enhance strength recovery during the third stage, the microstructure of the samples after low-temperature creep-aging in the third stage is presented in Figure 9b,d. It can be observed that the precipitates in both figures become denser compared to the previous stages. Notably, the precipitate sizes are predominantly distributed within the 35~40 nm and 110~120 nm ranges, exhibiting a distinct bimodal distribution. This phenomenon is attributed to the secondary precipitation of residual solute atoms after the high-temperature stage, resulting in the coexistence of newly formed fine phases and coarsened phases within the grains. Therefore, the low-temperature treatment in the third stage effectively promotes the secondary precipitation of some precipitates, significantly increasing their quantity, which contributes to the improvement of mechanical properties.

To further validate the observed precipitation behavior, we compared our findings with the existing literature. Previous studies have indicated [29] that introducing a new low-temperature CA stage prior to the conventional single-stage CA can effectively regulate the subsequent precipitation of the T1 phase. Specifically, moderately increasing the first-stage CA temperature weakens the promotion of GP zone formation, reducing the number of metastable precipitates. Consequently, a substantial amount of the T1 phase and a small quantity of the θ’ phase form during the first stage, significantly enhancing the thermal stability of the precipitates. This enables the microstructural features established in the first stage to be retained during the high-temperature stage, thereby mitigating the phenomenon of precipitate dissolution at elevated temperatures. Our experimental results (Figure 9e,c) are consistent with this conclusion, showing no significant difference in precipitate morphology between the two processes—155 °C-24 h-220 MPa and 155 °C-24 h-210 °C-3 h-220 MPa—effectively maintaining microstructural stability. Furthermore, to restore strength after high-temperature treatment, as shown in Figure 9b,d, the microstructure after the third-stage low-temperature CA exhibits denser precipitates compared to previous stages, indicating a certain degree of secondary precipitation. This phenomenon suggests an increase in the number of precipitates during the third stage. Such observations align with previous studies [39], which have demonstrated that the reprecipitation of new strengthening phases during the low-temperature stage can counteract the strength loss induced by high-temperature exposure. This specific aging sequence effectively regulates the evolution of precipitates, thereby influencing the deformation behavior and mechanical properties of the studied alloy.

## 4. Conclusions

In this study, the effect of aging temperatures at different stages on precipitates precipitated during multi-stage CA of Al-Cu-Li alloy was investigated by a combination of MD simulations and experimental tests, and the conclusions are summarized as follows:

(1) After undergoing the low-temperature-high-temperature-low-temperature three-stage CA process, the specimens achieved a tensile strength of 598 MPa and a yield strength of 559 MPa, which are comparable to the peak performance of the traditional single-stage CA specimens. This result demonstrates that the multi-stage CA process not only ensures a high degree of creep deformation but also maintains excellent mechanical properties, achieving a synergistic optimization of both high plasticity and high strength.
(2) When the temperature of the first-stage low-temperature CA is too low, it leads to the re-dissolution of precipitates during the high-temperature stage. Similarly, if the temperature of the second-stage high-temperature aging is too high, it will also cause the dissolution of a large amount of precipitates. Both scenarios result in a reduction in nucleation sites, thereby affecting the material’s performance.
(3) The segregation behavior of Li atoms promotes the precipitation of the T1 strengthening phase in Al-Cu-Li alloys. However, as the temperature increases, the segregation of Li atoms gradually diminishes, leading to the re-dissolution of the precipitate phase and consequently causing a deterioration in the material’s mechanical properties. The excellent agreement between the MD simulation results based on the NEP function and the experimental results further validates the reliability of the NEP function in simulating complex alloy systems.

## Figures and Tables

**Table 1:** Boundary condition settings for MD simulations.

Boundary Condition	Parameters
Model size	181.61 Å × 105.92 Å × 159.78 Å
Boundary conditions (X, Y, Z)	P, P, P (P: Periodic boundary conditions)
Simulation pressure	220 MPa
Simulation temperature	90, 155, 210, 260 °C
Time step	1 fs
Simulation steps	2,000,000

**Table 2:** The main chemical compositions of Al-Cu-Li alloys (wt.%).

Cu	Li	Mg	Ag	Fe	Zr	Mn	Si	Ti	Al
4.18	1.19	0.40	0.38	0.052	0.094	0.0047	0.014	0.018	Bal.

## Data Availability

The original contributions presented in this study are included in the article. Further inquiries can be directed to the corresponding author(s).

## References

[1] Dursun T., Soutis C. (2014). Recent developments in advanced aircraft aluminium alloys. Mater. Des. (1980–2015).

[2] Honma T., Yanagita S., Hono K., Nagai Y., Hasegawa M. (2004). Coincidence Doppler broadening and 3DAP study of the pre-precipitation stage of an Al–Li–Cu–Mg–Ag alloy. Acta Mater..

[3] Zhang L., Heng L.I., Tianjun B.I.A.N., Changhui W.U., Yanfeng Y.A.N.G. (2025). Significant reduction of anisotropy in stress relaxation aging and mechanical properties improvement for 2195 Al-Cu-Li alloy subjected to plastic loading. Chin. J. Aeronaut..

[4] Long S., Jiang Y.P., Xia R.Z., Peng P., Zhang C., Li S.S., Dai Q.W., Zhou J. (2024). Recrystallization behavior and kinetic analysis of an Al-Cu-Li alloy during hot deformation and subsequent heat treatment. Mater. Charact..

[5] Song L., Li H., Zhao G., Bao X., Zheng Z. (2024). Quantification of precipitate evolution in cast Al–Li alloys containing Cu and Mg additions using small-angle X-ray scattering. J. Mater. Res. Technol..

[6] Gao Z., Chen J.H., Duan S.Y., Yang X.B., Wu C.L. (2016). Complex precipitation sequences of Al-Cu-Li-(Mg) alloys characterized in relation to thermal ageing processes. Acta Metall. Sin. Engl. Lett..

[7] Duan S., Liu Z., Guo F., Pan Y., Matsuda K., Zou Y. (2023). Precipitates evolution during artificial aging and their influence on mechanical properties of a cast Al–Cu–Li alloy. J. Mater. Res. Technol..

[8] Deschamps A., Decreus B., De Geuser F., Dorin T., Weyland M. (2013). The influence of precipitation on plastic deformation of Al–Cu–Li alloys. Acta Mater..

[9] Dorin T., Deschamps A., De Geuser F., Sigli C. (2014). Quantification and modelling of the microstructure/strength relationship by tailoring the morphological parameters of the T1 phase in an Al–Cu–Li alloy. Acta Mater..

[10] Xie B., Huang L., Xu J., Su H., Zhang H., Xu Y., Li J., Wang Y. (2022). Effect of the aging process and pre-deformation on the precipitated phase and mechanical properties of 2195 Al–Li alloy. Mater. Sci. Eng. A.

[11] Hu W., Chen J., Xu J., Ren J., Miao J., Xing T., Guan R., Ojo O.A. (2023). Relationship between precipitation behavior and loading orientations of the creep-aged Al–Cu–Li single crystal. J. Mater. Res. Technol..

[12] Wu C.H., Li H., Lei C., Zhang D., Bian T.J., Zhang L.W. (2023). Origin and effect of anisotropy in creep aging behavior of Al–Cu–Li alloy. J. Mater. Res. Technol..

[13] Wang D., Zhan L., Liu C., Zeng Q., Xu Y., Ma B., Gan K., Lai R., Li Y., Liu C. (2024). Study on creep aging behavior of the friction stir welded Al-Li alloy joints under different stresses. Mater. Charact..

[14] Al-Motasem A.T., Posselt M., Bergner F., Birkenheuer U. (2011). Structure, energetics and thermodynamics of copper–vacancy clusters in bcc-Fe: An atomistic study. J. Nucl. Mater..

[15] Molnar D., Mukherjee R., Choudhury A., Mora A., Binkele P., Selzer M., Nestler B., Schmauder S. (2012). Multiscale simulations on the coarsening of Cu-rich precipitates in α-Fe using kinetic Monte Carlo, molecular dynamics and phase-field simulations. Acta Mater..

[16] Clouet E., Barbu A., Laé L., Martin G. (2005). Precipitation kinetics of Al3Zr and Al3Sc in aluminum alloys modeled with cluster dynamics. Acta Mater..

[17] Yi D., Wei G. (2024). MD Simulation of Diffusion Behaviors in Collision Welding Processes of Al-Cu, Al-Al, Cu-Cu. Comput. Mater. Contin..

[18] Ouyang D., Mao R., Zhang L., Liang S., Song J. (2024). Study on the tensile properties of Al-Zn-Mg alloy based on molecular dynamics. Eng. Fail. Anal..

[19] Li F., Dong T., Zhang C., Chen G., Chen S., Chen K., Zhu C. (2024). Controllable precipitation behavior near grain boundaries enabled by artificial strain concentration in age-hardening aluminum alloys. Mater. Sci. Eng. A.

[20] Tang L., Guo A., Farid W., Wang Z., Kong C., Zhang L., Yu H. (2025). Enhanced mechanical and corrosion properties of 2195 Al-Li alloy via cryogenic pre-rolling and aging. J. Alloys Compd..

[21] Shuai X., Hong M.A.O., Sai T.A.N.G., Yi K.O.N.G., Yong D.U. (2025). Atomic-scale insights into microscopic mechanisms of grain boundary segregation in Al−Cu alloys. Trans. Nonferrous Met. Soc. China.

[22] Liao H., Du J.P., Kimizuka H., Ogata S. (2025). Atomistic simulation of Guinier–Preston zone nucleation kinetics in Al–Cu alloys: A neural network-driven kinetic Monte Carlo approach. Comput. Mater. Sci..

[23] Xu L., Tong C., Zhan L., Xu Y., Liu C., Huang M., Yang Y., Ma B., Wang Y. (2022). Improved creep forming efficiency and retained performance via a novel two-stage creep aging process of Al–Zn–Mg–Cu alloys. Mater. Sci. Eng. A.

[24] Chen F., Yu Y., Jiang Y., Zeng Q., Zhan L. (2025). A new multi-stage creep aging process for improving creep age formability and maintaining performance of Al-Li alloy. Prog. Nat. Sci. Mater. Int..

[25] Fan Z., Wang Y., Ying P., Song K., Wang J., Wang Y., Zeng Z., Xu K., Lindgren E., Rahm J.M. (2022). GPUMD: A package for constructing accurate machine-learned potentials and performing highly efficient atomistic simulations. J. Chem. Phys..

[26] Stukowski A. (2009). Visualization and analysis of atomistic simulation data with OVITO–the Open Visualization Tool. Model. Simul. Mater. Sci. Eng..

[27] Chen F., Wang H., Jiang Y., Zhan L., Yang Y. (2025). Development of a Neuroevolution Machine Learning Potential of Al-Cu-Li Alloys. Metals.

[28] Fan Z., Zeng Z., Zhang C., Wang Y., Song K., Dong H., Chen Y., Ala-Nissila T. (2021). Neuroevolution machine learning potentials: Combining high accuracy and low cost in atomistic simulations and application to heat transport. Phys. Rev. B.

[29] Chen F., Wang P., Zhan L., Ruan X., Wu H., Xu Y., Ma B., Liu C., Zeng Q., Hu Z. (2022). Creep behavior and microstructure evolution during two-stage creep aging of a 2195 Al–Li alloy. Mater. Sci. Eng. A.

[30] Wang K., Zhang C., Cheng Z., Zhao H., Meng Z., Chen L., Zhao G. (2024). Dynamic evolution of the T1 phase and its effect on continuous dynamic recrystallization in Al–Cu–Li alloys. Int. J. Plast..

[31] Wu M., Yao T., Xiao D., Yuan S., Li Z., Wang J., Huang L., Liu W. (2024). Precipitation behavior and properties of an Al-8.26 Zn-1.95 Mg-1.89 Cu-0.08 Sc-0.17 Zr alloy with different dislocation morphologies via pre-treatment. J. Mater. Res. Technol..

[32] Xie Y., Liu S., Guo X., He X., Liang C., Deng Y. (2025). Modulation of precipitation behavior by dislocations and alloying for superior strength-ductility balance in Al-Cu-Li alloys. J. Alloys Compd..

[33] Wang X.M., Li G.A., Jiang J.T., Shao W.Z., Zhen L. (2019). Influence of Mg content on ageing precipitation behavior of Al-Cu-Li-x alloys. Mater. Sci. Eng. A.

[34] Wang J., Yang K., Lu Y., Cheng X., You D., Zhou D., Wang M., Zhu B., Li J. (2024). Investigation on the microstructural evolution and corrosion behaviors of Zn-microalloyed Al-Cu-Li alloys. Mater. Today Commun..

[35] Deng Y., Bai J., Wu X., Huang G., Cao L., Huang L. (2017). Investigation on formation mechanism of T1 precipitate in an Al-Cu-Li alloy. J. Alloys Compd..

[36] Li J.F., Huang J.L., Liu D.Y., Chen Y.L., Zhang X.H. (2019). Distribution and evolution of aging precipitates in Al-Cu-Li alloy with high Li concentration. Trans. Nonferrous Met. Soc. China.

[37] Firrao D., Scavino G., Doglione R. (1989). Precipitation phenomena during an Al-Li-Cu alloy ageing. Proceedings of the 5th International Conference on Aluminum-Lithium Alloys.

[38] Engler O., Marioara C.D., Aruga Y., Kozuka M., Myhr O.R. (2019). Effect of natural ageing or pre-ageing on the evolution of precipitate structure and strength during age hardening of Al–Mg–Si alloy AA 6016. Mater. Sci. Eng. A.

[39] Xue L.W., Jia H.L., Wang J.K., Zha M., Jin S.B., Wang H.Y. (2025). Superior strength-ductility synergy of Al-Si-Cu-Mg alloys achieved by regulating solute clusters and precipitates: Experimental validation and numerical simulation. Int. J. Plast..

[40] Yu Z., Li H., Cai P., Fu X., Feng Z., Zhang L., Wang J., Xiao N. (2023). Effect of aging route on the precipitation behavior and thermal stability of Al–Cu–Mg–Ag alloy. J. Mater. Res. Technol..

